# Supplementary material for: Memory effect assisted imaging through multimode optical fibres
Source: Nat Commun. 2021 Jun 18;12:3751. doi: 10.1038/s41467-021-23729-1 (PMC8213736; doi:10.1038/s41467-021-23729-1)
Supplement: Supplementary file 1 — Supplementary Information [file 41467_2021_23729_MOESM1_ESM.pdf]

Memory effect assisted imaging through multimode optical fibres: supplementary information

Shuhui Li,[1, 2, *] Simon A. R. Horsley,[1] Tomáš Tyc,[3] Tomáš Čižmár,[4, 5] and David B. Phillips[1,†]

[1]Physics and Astronomy, University of Exeter, Exeter, EX4 4QL. UK.

[2]Wuhan National Laboratory for Optoelectronics, Huazhong University of Science and Technology, Wuhan 430074, Hubei, China.

[3]Department of Theoretical Physics and Astrophysics, Masaryk University, Kotlarska 2, 61137 Brno, Czech Republic.

[4]Leibniz Institute of Photonic Technology, Albert-Einstein-Straße 9, 07745 Jena, Germany.

[5]Institute of Scientific Instruments of CAS, Kr'alovopolská 147, 612 64, Brno, Czech Republic.

[*]Electronic address: shli@hust.edu.cn

[†]Electronic address: d.phillips@exeter.ac.uk

Supplementary Note 1: Disruption of the 2D shift-shift memory effect in MMFs

Here we analyse why the 2D shift-shift memory effect is disrupted in MMFs. We start by considering propagation through a block of glass of permitivity ε , finite thickness d , but infinite area. In this case the system trivially possesses a 2D shift–shift memory effect, *and* a rotational memory effect that holds about any axis. The transmission matrix for propagating from the entrance surface to the exit surface is given by

$$\mathbf{T} = \mathcal{F}^{-1}\mathrm{diag}\left[\mathrm{e}^{\mathrm{i}\mathbf{k}_z d}\right]\mathcal{F}\,, \tag{1}$$

where the component of the wave-vector k normal to the glass-air interface is $k_z = \sqrt{\varepsilon k_0^2 - k_x^2 - k_y^2} = \sqrt{\varepsilon k_0^2 - k_r^2}$. This transmission matrix exhibits two memory effects. The first is the shift-shift memory effect. A spatial shift of the input field by $(\Delta x, \Delta y)$ can be written in terms of a phase shift sandwiched between a Fourier transform and its inverse

$$\boldsymbol{O} = \mathcal{F}^{-1}\mathrm{diag}\left[\mathrm{e}^{-\mathrm{i}(\mathbf{k}_x\Delta x + \mathbf{k}_y\Delta y)}\right]\mathcal{F}\,. \tag{2}$$

Given that $\boldsymbol{O}$ and $\mathbf{T}$ share the same basis, they commute. As a consequence, a spatial translation of the input field results in the same translation of the output. Instead of the 2D Fourier transform, we can also perform an angular spectral decomposition in cylindrical coordinates, which in this case is equivalent to the Fourier transform (Supplementary Equation 1). If the input field is $\mathbf{u}$, the transform $\mathbf{U}^{-1}$ into the radial wave-vector k_r and angular momentum ℓ variables is given by

$$\mathbf{U}^{-1}\mathbf{u} = \int_0^{2\pi}\frac{d\theta}{2\pi}\int_0^{\infty} r dr \mathcal{J}_\ell(k_r r)\mathrm{e}^{-\mathrm{i}\ell\theta}u(r,\theta)\,. \tag{3}$$

The transmission matrix is also diagonal in this basis

$$\mathbf{T} = \mathbf{U}\mathrm{diag}\left[\mathrm{e}^{\mathrm{i}\mathbf{k}_z d}\right]\mathbf{U}^{-1} \tag{4}$$

and analogously the rotation of the field by the angle $\Delta\theta$ corresponds to the operator $\boldsymbol{O} = \mathbf{U}\mathrm{diag}\left[\mathrm{e}^{-\mathrm{i}\ell\Delta\theta}\right]\mathbf{U}^{-1}$. Given that the rotation operator and the transmission matrix (Supplementary Equation 4) commute, a rotation of the input field about any axis results in the same rotation of the output field.

Now consider the case of a MMF. The only change to our glass block is the introduction of a cylindrical boundary centered around the origin $r = 0$. The modes are now given by Equation 4 in the main text, and the transformation into the basis of the transmission matrix is now modified from Supplementary Equation 3 to

$$\mathbf{U}^{-1}\mathbf{u}=\int_0^{2\pi}\frac{d\theta}{2\pi}\int_0^{\infty}rdr\psi_{\ell,p}(r,\theta)u(r,\theta)=\int_0^{2\pi}\frac{d\theta}{2\pi}\int_0^{\infty}rdrN_{\ell,p}e^{i\ell\theta}u(r,\theta)\begin{cases}\mathcal{J}_\ell\left(u_{\ell,p}r/a\right)/\mathcal{J}_\ell\left(u_{\ell,p}\right) & \text{for } r<a\\ \mathcal{K}_\ell\left(\omega_{\ell,p}r/a\right)/\mathcal{K}_\ell\left(\omega_{\ell,p}\right) & \text{for } r\geq a,\end{cases} \quad (5)$$

The angular part of the transformation shown in Supplementary Equation 5 is unmodified from the case of the infinite glass block (see Supplementary Equation 3), as long as rotation is about the origin, and therefore the rotational memory effect persists, where a rotation of the input results in a similar rotation of the output. However, the effect of the boundary is to constrain the value of k_r to discrete values that depend on the angular momentum ℓ. This modification to Supplementary Equation 3 means that the transmission matrix can no longer be diagonal in the Fourier basis as it was in Supplementary Equation 1. This is to be expected because of the broken translation symmetry. Thus the shift-shift memory effect is destroyed. We note that for short pieces of low NA fibre with large core diameters, we would expect a remnant of the shift-shift memory to persist - simply by virtue of the fact that in this case some fields may propagate through the fibre without interacting with the core-cladding interface, i.e. these fields propagate exactly as in the block of glass of infinite area described above.

The lack of a ‘radially shifting’ memory effect in MMFs means that we require knowledge of the full TM of the system to move a point radially. This can be shown as follows: We denote the output field focussed onto the guide-star as $\mathbf{v}^{m_0}$, which has all elements equal to zero except for one at the spatial point corresponding to the location of the guide-star, indexed by m_0. To shift a focus at the distal facet from the point m_0 in real-space to another point m_0', the modulation $\boldsymbol{O}'$ that must be applied to the input field $\mathbf{u}^{\text{gs}}$ is given by

$$\boldsymbol{O}'=\mathbf{T}^{-1}\mathbf{\Pi}_{m_0}^{m_0'}\mathbf{T}=\ \mathbf{P}(\mathbf{D}^{\dagger}\mathbf{P}^{\dagger}\mathbf{\Pi}_{m_0}^{m_0'}\mathbf{P}\mathbf{D})\mathbf{P}^{\dagger}, \quad (6)$$

where $\mathbf{\Pi}_{m_0}^{m_0'}$ is a permutation matrix that swaps the m_0 and m_0' elements of a vector. $\mathbf{\Pi}_{m_0}^{m_0'}$ is constructed by modifying an $N\times N$ identity matrix (where N is the total number of pixels across the fibre facet, i.e. the number of rows in $\mathbf{P}$). The modifications are that the diagonal elements (m_0,m_0) and (m_0',m_0') are replaced with 0, and off-diagonal elements (m_0',m_0) and (m_0,m_0') are replaced with 1. The intuition behind the form of $\boldsymbol{O}'$ is that it propagates $\mathbf{u}^{\text{gs}}$ to the output facet, swaps the location of the focus to m_0', and propagates the resulting field back to the proximal facet. The brackets in the above expression indicate that the analogue of the diagonal matrix $\mathbf{M}$ introduced through Equation 1 in the main text is here given by

$$\mathbf{M}'=\ \mathbf{D}^{\dagger}\mathbf{P}^{\dagger}\mathbf{\Pi}\mathbf{P}\mathbf{D}. \quad (7)$$

Formulating $\boldsymbol{O}'$ in this way highlights that because there is no conventional memory effect, the modulation required to shift the focus radially is not diagonal, and requires knowledge of not just the basis in which the TM is diagonal, but also the diagonal elements of $\mathbf{D}$ - i.e. knowledge of the TM itself.

Supplementary Note 2: The quasi--radial memory effect in MMFs

In this section we consider how a modulation of the input field at one end of an MMF can modify the field at the output in a deterministic way. As an example we consider, as in Equations 11-15 of the Methods in the main text, a step-index MMF. This has modes given by Equation 4 of the main text. As indicated in the Methods, the elements of the matrix $\mathbf{P}$ are given by

$$P_{nm}=\psi_{\ell_m,p_m}(r_n,\theta_n) \quad (8)$$

where m and n are indices that are arranged to run through the pair of spectral indices ℓ,p and the Cartesian grid of coordinates r,θ respectively. The normalization of the modes is chosen such that $\mathbf{P}\mathbf{P}^{\dagger}=\mathbf{1}$. As stated in the main text, the

transmission matrix is of the form $\mathbf{T} = \mathbf{PDP}^{\dagger}$, and thus the modulations of the input field that modify the output field in the same manner are of the form $\boldsymbol{O} = \mathbf{PMP}^{\dagger}$ where $\mathbf{M}$ is a diagonal matrix. We now examine what is possible in the basis of a MMF. With the indices made explicit, the transformation of the input mode is given by

$$O_{nm} = \sum_q P_{nq} M_q P_{mq}^{\leftarrow} = \sum_q M_q \psi_{\ell_q, p_q}(r_n, \theta_n) \psi_{\ell_q, p_q}^{\leftarrow}(r_m, \theta_m). \tag{9}$$

For general M_q this transformation will have quite a complicated effect on the output field. However we can find several relatively simple transformations that have interesting deterministic effects on the field. Here we describe three of these transformations:

(i) The main text gives an example of one possible transformation: a modulation based on the radial mode index p on the input beam: $\mathbf{M} = \mathrm{diag}[e^{i\mathbf{p}\delta\rho}]$, i.e. a phase ramp linearly proportional to p, where vector $\mathbf{p}$ holds the radial index of each PIM, and where $\delta\rho = \pi\delta r / a$, and δr is radial distance across the fibre facet. As shown in Fig. 2 of the main text, this modulation moves the intensity of a focussed point radially, but also spreads it out into a variety of patterns which are dependent on the starting position of the focus.

(ii) It is possible to improve on (i) - i.e. move the focus radially while spreading it out slightly less - with knowledge of the radius of the initial location of the point, r_0. To understand how best to change the phase of each PIM, we need to know how quickly the phase changes with the radius r. However, in the radial direction the modes are standing waves, so changing the phase will not move the point only in the desired direction, but will create another copy of the point moving in the opposite direction (see below). In any case, the total wave-number in the fibre, i.e. $k_0 n_{\mathrm{core}}$ (symbols defined in Methods) has to be divided between the z -direction (where the wavenumber is the propagation constant β), the angular direction (where the local wavenumber at radius r is ℓ / r), and the radial direction (where we denote the wavenumber of the nth PIM by $k_r(n)$). For the nth PIM this gives

$$k_0^2 n_{\mathrm{core}}^2 = k_r(n)^2 + \beta_n^2 + \frac{\ell_n^2}{r^2}, \tag{10}$$

from which we find

$$k_r(n, r) = \sqrt{k_0^2 n_{\mathrm{core}}^2 - \beta_n^2 - \frac{\ell_n^2}{r^2}}. \tag{11}$$

Here, by writing $k_r(n, r)$, we emphasise that the radial wavenumber depends on both the PIM and the radius. The point r_t for which $k_r(n, r_t) = 0$ is the radial turning point where the radial part changes from evanescent (for $r < r_t$) to oscillatory (for $r \geq r_t$). In most of the evanescent region the wave is very weak, so in case that k_r turns out to be imaginary, we use no correction. Therefore, if we want to shift a focussed spot at r_0 to a new position r_1, the simplest correction would be to add the phase $\phi_n = (r_1 - r_0) k_r(n, r)$. However, it turns out to be better to add the phase

$$\phi_n = (r_1 - r_0) k_r\left(n, \frac{r_0 + r_1}{2}\right), \tag{12}$$

to the nth PIM, i.e. to evaluate the radial wavenumber half-way between the original and desired positions. Supplementary Figure 1 shows the behaviour of this quasi-radial memory effect.

(iii) A third possible transformation is to split an annular ring of light of radius r (i.e. with no azimuthal variation) into two annuli that move radially away from one another. To achieve this we assume the initial field at the end of the MMF is concentrated to a diffraction limited annulus away from the origin. With this assumption we can approximate the

Bessel functions describing the PIMs as the first term in their asymptotic expansion, see Eq. 10.17.3 of [1].

$$J_{\ell_q}\left(u_{\ell_q,p_q} r_n / a\right) \sim \left(\frac{2a}{\pi u_{\ell_q,p_q} r_n}\right)^{\frac{1}{2}} \cos\left(u_{\ell_q,p_q} r_n / a - \frac{1}{2}\ell_q \pi - \frac{1}{4}\pi\right),$$
$$Y_{\ell_q}\left(u_{\ell_q,p_q} r_n / a\right) \sim \left(\frac{2a}{\pi u_{\ell_q,p_q} r_n}\right)^{\frac{1}{2}} \sin\left(u_{\ell_q,p_q} r_n / a - \frac{1}{2}\ell_q \pi - \frac{1}{4}\pi\right). \tag{13}$$

In this asymptotic form, the Bessel functions are approximately a cosine function divided by the square root of the radius. Multiplying Supplementary Equation 13 by the modulation factor $\cos(u_{\ell_q,p_q}\delta r / a)$ and applying the double angle formulae $\cos(A)\cos(B) = [\cos(A+B)+\cos(A-B)]/2$ and $\sin(A)\cos(B) = [\sin(A+B)+\sin(A-B)]/2$, we have

$$J_{\ell_q}\left(u_{\ell_q,p_q} r_n / a\right) \mathrm{e}^{\mathrm{i}u_{\ell_q,p_q}\delta r/a} \sim \frac{1}{2}\left[\sqrt{\frac{r_n+\delta r}{r_n}} J_{\ell_q}(u_{\ell_q,p_q}(r_n+\delta r)/a) + \sqrt{\frac{r_n-\delta r}{r_n}} J_{\ell_q}(u_{\ell_q,p_q}(r_n-\delta r)/a)\right]. \tag{14}$$

Therefore making the choice of modulation $M_q = \cos(u_{\ell_q,p_q}\delta r / a)$ and substituting Supplementary Equation 14 into Supplementary Equation 9 we have

$$O_{nm} \sim \frac{1}{2}\left[\sqrt{\frac{r_m}{r_{m-\Delta n}}}\delta_{n+\Delta n,m} + \sqrt{\frac{r_m}{r_{m+\Delta n}}}\delta_{n-\Delta n,m}\right], \tag{15}$$

where δr moves the point radially by approximately Δn points on the grid. Therefore in a step--index MMF there is a quasi radial memory effect where a annular ring can be split, translating one copy inwards radially (and increasing the intensity by $\sqrt{r_m / r_{m-\Delta n}}$), and one outwards (decreasing the intensity by $\sqrt{r_m / r_{m+\Delta n}}$). Supplementary Figure 2, top row, shows this quasi-radial memory effect. Intriguingly, we also found that this same transformation expanded individual points about their initial positions, as shown in Supplementary Figure 2, lower three rows.

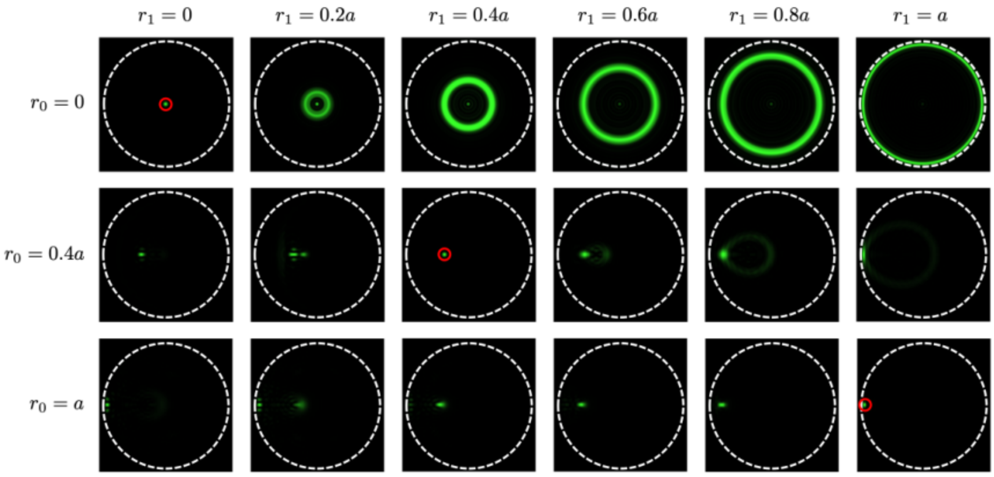

Supplementary Figure 1: Quasi-radial memory effect based on radial wave-vector: Transformation of focussed spots (initial locations marked with red circles) in the radial direction using Eqn. s10.

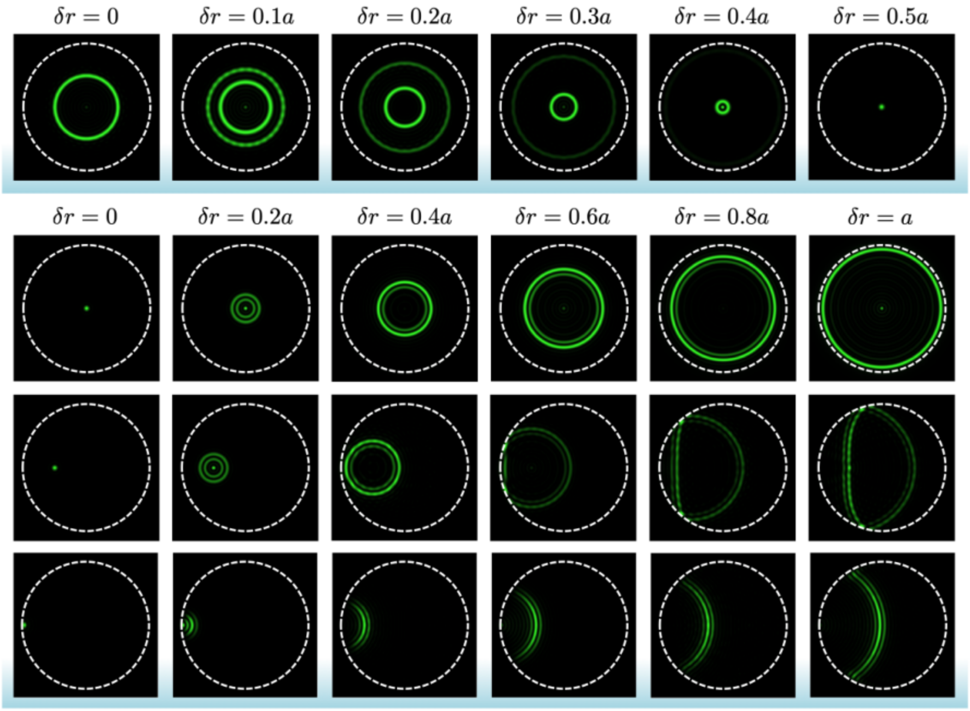

Supplementary Figure 2: **Quasi-radial memory effect based on normalised transverse wave-number**: Top row: transformation of a diffraction-limited annular ring of light (initial field left most panel) into two rings moving in opposite directions. Lower three rows: transformation of focussed spots (initial locations left most panels) radially about their own initial location. In the second from bottom row the field appears to 'reflect' from the core-cladding boundary.

Supplementary Note 3: Experimental set-up

The transmission matrix measurement set-up is shown in detail in Supplementary Figure 3. It is based on a Mach-Zehnder interferometer, using a linearly polarised 1mW HeNe laser of wavelength 633 nm. Half wave-plate HW1 and polarising beam splitter PBS1 enable the intensity of the laser to be modulated - by rotating the half wave plate. Unwanted light is sent to the beam block. HW2 and PBS2 enable the relative intensity of light in the signal and reference arms of the interferometer to be balanced. The laser beam transmitted into the signal arm is spatially filtered to improve the beam quality (SF1), and expanded to fill the chip of a digital micro-mirror device (DMD) (ViALUX V-7001, resolution of 1024×768, modulation rate up to 22.727 kHz). The light diffracting from the DMD is spatially filtered (SF2), blocking all but the 1st diffraction order. Using this set-up the appropriate choice of binary hologram on the DMD can create a beam of arbitrary spatially varying amplitude and phase (up to a maximum spatial frequency) in the Fourier plane of the DMD. The Fourier plane of the DMD is imaged onto the proximal facet of a step index multimode fibre (MMF) ($\sim$ 30 cm in length, core diameter 50 μm , 0.22 NA, supporting 754 modes per polarization at a wavelength of 633 nm). The distal facet of the MMF is imaged onto a high-speed camera (Camera 1: Basler Pilot GigE, resolution 648×488, full-sensor frame-rate up to 210 Hz, reduced region of interest frame-rate up to 400 Hz) that is electronically synchronised with the DMD (here the DMD is set to trigger the camera when the DMD displays a new pattern). Quarter wave-plate QWP1 transforms the linearly polarized beam to a circularly polarised beam before the MMF. The cylindrical symmetry of the MMF means that the circular polarisation of the light is largely preserved during transmission through the MMF. The objective lenses at either end of the fibre are mounted on 3D translation stages (not shown) to enable alignment with the ends of the MMF. QWP2 after the MMF transforms the output field back to linear polarisation. Light transmitted into the reference arm of the interferometer is sent through a polarisation maintaining single mode fibre, with half wave-plates at

each end (HW3 and HW4) to control the orientation of the linear polarisation. The optical path lengths of the signal and reference arms are equivalent to within the coherence length of the laser (~ 10 cm). The reference beam is then combined with the signal beam using a beam splitter (BS2), and together they are imaged onto the high-speed camera. The alignment cameras 2 and 3 (also Basler Pilot GigE) are in the image plane of the proximal facet of the MMF. Camera 2 images the incident laser beam, enabling aberration correction of the optical set-up before the MMF (see below). To align the MMF in the system, a red LED illuminator is used to flood illuminate the proximal facet of the fibre. LED light and/or laser light reflected from the proximal facet is imaged onto camera 3, enabling fibre position to be adjusted until both the fibre-facet and the laser spot are in focus simultaneously. LED illumination transmitted through the MMF and exiting the distal fibre facet is used to ensure the image of the distal facet is in focus on camera 1.

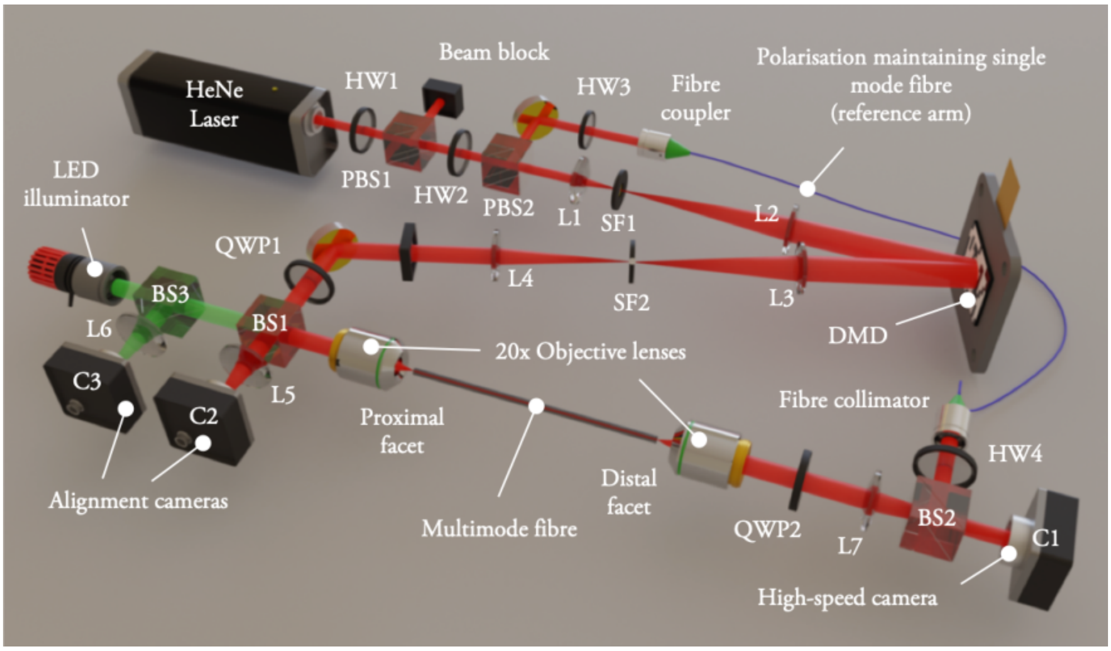

Supplementary Figure 3: **Schematic of optical setup**: Experimental set-up to measure the full TM of a MMF, emulate ATM measurement with a guide-star, and image through the MMF. HW = half wave-plate, QW = quarter wave-plate, BS = beamsplitter, PBS = polarizing beamsplitter, SF = spatial filter. Focal lengths of lenses 1-7 are: L1 = 50 mm, L2 = 400 mm, L3 = 300 mm, L4 = 200 mm, L5 = 500 mm, L6 = 600 mm, & L7 = 150 mm.

Supplementary Note 4: TM and ATM measurement and control of output field

Before the TM of the MMF is measured, aberrations in the beam path up to the input to the MMF are first characterised using the super-pixel based in-situ wavefront correction methods described in [2], using the DMD synchronised with the alignment camera at 100 Hz. This enables calculation of an aberration correcting phase function that counteracts any significant aberrations in the field generated at the input of the MMF.

To fully sample the TM of the MMF, we use phase stepping holography with an external reference beam provided by the reference arm of the interferometer. A grating applied to the DMD (incorporating the aberration correcting phase pattern from above) raster scans a focussed spot over a Cartesian grid of points on the proximal facet of the fibre. At each location we record four output images: each with the a relative phase shift of the focussed spot (with respect to the external reference) of $\zeta = 0$, $\pi / 2$, π , and $3\pi / 2$ rad. (in each case achieved by adding the appropriate global phase shift to the input function $\mathcal{B}$ used to create the DMD hologram). These phase shifts can be stored in a column vector $\boldsymbol{\zeta}$. Each high-speed camera pixel images the interference of a point in the field at the distal facet of the fibre with the external reference beam, which can be stored as a column vector of four intensity measurements, $\mathbf{q}$. The phase $\theta_{\text{dist}}(g,h)$ of the distal field,

at point g, h (denoting the camera pixel row and column respectively), is then given by $\theta_{\text{dist}} = \arg[\mathbf{s} \cdot \mathbf{q}]$, where the nth element of vector $\mathbf{s}$ is given by $s(n) = \sin(\zeta(n))$. The amplitude $A(g, h)$ of the field at point g, h, can be calculated directly as the square root of the intensity image of the field with the external reference beam turned off - which is performed once all of the phase measurements for all of the input modes have been captured. To mitigate phase drift between the signal and reference arms of the interferometer during TM characterisation, the measurements are interlaced with a standard measurement allowing phase drift to be tracked and subtracted.

A focussed spot at the nth location on the proximal facet, generates the field $A_n e^{i\theta_{\text{dist},n}}$ which is measured as described above. $A_n e^{i\theta_{\text{dist},n}}$ is then reshaped into a column vector, which forms the n th column of $\mathbf{T}_{\text{sp}\mapsto\text{px}}$ where here the subscripts indicate the transmission matrix of the MMF is measured in the input basis of spots and output basis of camera pixels. Experimentally the input and output bases are different due to the differing resolution of the camera at the output from the focussed spot sizes and separations at the input. In order to transform the measured TM into the quasi--diagonal PIM basis, $\mathbf{D}_{\text{pm}\mapsto\text{pm}}$, we follow the coarse alignment methods described in ref. [3], and calculate

$$\mathbf{D}_{\text{pm}\mapsto\text{pm}} = \mathbf{P}^{\dagger}_{\text{pm}\mapsto\text{px}} \mathbf{R}_{\text{dl}} \mathbf{T}_{\text{sp}\mapsto\text{px}} \mathbf{R}^{\dagger}_{\text{pr}} \mathbf{P}_{\text{pm}\mapsto\text{sp}} = \mathbf{P}'^{\dagger}_{\text{px}\mapsto\text{pm}} \mathbf{T}_{\text{sp}\mapsto\text{px}} \mathbf{P}'_{\text{sp}\mapsto\text{pm}}, \tag{16}$$

where $\mathbf{P}_{\text{pm}\mapsto\text{px}}$ and $\mathbf{P}_{\text{pm}\mapsto\text{sp}}$ are numerically calculated as described in the Methods, using knowledge of the camera pixel pitch and the magnification with which the distal facet is imaged onto the camera, and the diffraction limit and spot separation chosen at the input. $\mathbf{R}_{pr}$ and $\mathbf{R}_{dl}$ are misalignment operators equivalent to those described in the Methods, that here can be found directly from the fully sampled TM $\mathbf{T}_{\text{sp}\mapsto\text{px}}$ itself, as described in [3]. In the final equation on the right hand side the misalignment matrices have been absorbed into the input and output basis transformations which are now denoted by $\mathbf{P}'_{\text{pm}\mapsto\text{px}}$ and $\mathbf{P}'^{\dagger}_{\text{pm}\mapsto\text{sp}}$. We note that in this work we did not perform optimisation of fibre parameters themselves as carried out in [3], however this provides a route to significantly improving the power on the diagonal of $\mathbf{T}_{\text{pm}\mapsto\text{pm}}$. Supplementary Figure 4 shows an example of an experimentally measured TM in the PIM basis - this is an enlarged version of Figure 4b shown in the main text.

Calculation of the ATM proceeds following Equations 7-9 of the main text. Experimentally, the ATM is given by

$$\mathbf{T}'_{\text{sp}\mapsto\text{px}} = \mathbf{P}'_{\text{pm}\mapsto\text{px}} \mathbf{D}'_{\text{pm}\mapsto\text{pm}} \mathbf{P}'^{\dagger}_{\text{pm}\mapsto\text{sp}}, \tag{17}$$

where $\mathbf{D}'_{\text{pm}\mapsto\text{pm}}$ is calculated as described in the main text. To control the field at the distal facet, we actually need to calculate the inverse of the ATM which is given by

$$\mathbf{T}'^{-1}_{\text{sp}\mapsto\text{px}} = \mathbf{P}'_{\text{pm}\mapsto\text{sp}} \mathbf{D}'^{*}_{\text{pm}\mapsto\text{pm}} \mathbf{P}'^{\dagger}_{\text{pm}\mapsto\text{px}}, \tag{18}$$

where the * operation indicates the complex conjugate. An input field $\mathbf{u}$ (in the spot basis) required to create a given output field $\mathbf{v}$ (in the pixel basis), such as a focussed or defocussed spot at an arbitrary location on the distal facet, is then calculated using $\mathbf{u} = \mathbf{T}'^{-1}_{\text{sp}\mapsto\text{px}} \mathbf{v}$. Once $\mathbf{u}$ is obtained, we then calculate the field required on the DMD to generate $\mathbf{u}$ at the

proximal facet of the fibre, and encode this field in the DMD hologram as described in Methods.

Measurement of the phase of PIMs that have a smaller overlap with the guide-star suffer from higher noise levels, and so we also explored a method to suppress contributions from this noise in the final inverse ATM used to calculate the required patterns. In this case we artificially reduced the amplitude of the complex diagonal elements of $\mathbf{D}'^*$ in proportion to their level of overlap with the guide-star. This had the effect of reducing the power of PIMs that we had not measured as well in the final field and gave a small improvement to the output field fidelities.

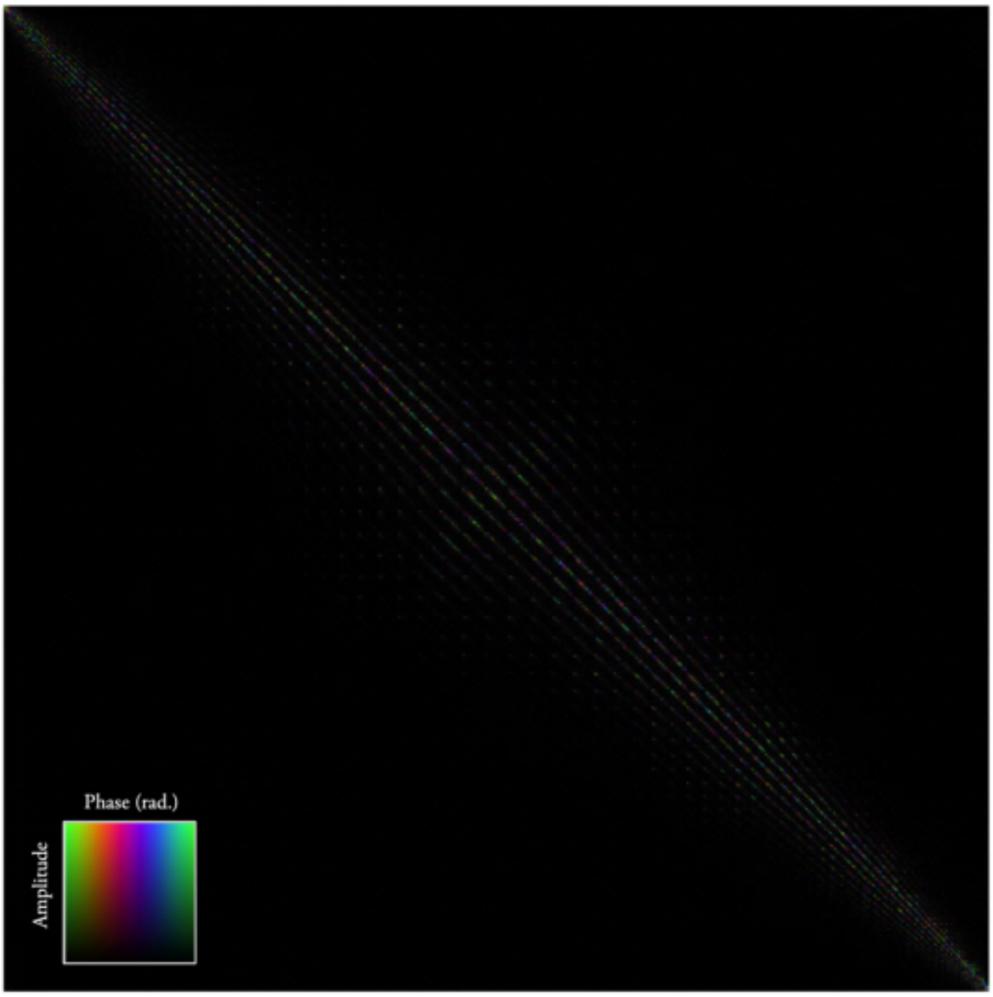

Supplementary Figure 4: **MMF TM**: Fully sampled experimentally measured TM (right handed circular polarisation input and output only) of a MMF, supporting 754 modes per polarisation at 633 nm, represented in the PIM basis.

Supplementary Note 5: Refocusing spots created at the distal facet of a MMF

In order for the resolution target to be imaged in our experiment, it is necessary to refocus the focus ~ 40 μm beyond the distal facet of the fibre. This is achieved by adding a focusing phase term (i.e. a Fresnel lens phase function) to the hologram displayed on the DMD. This follows from the assumption that the TM the MMF is diagonal in the PIM basis, and so preserves the radial component of the k-vector of transmitted light. Supplementary Figure 5 shows modeling (a-c) and experimental results (d-f) demonstrating the refocusing of spots and arrays of spots away from the distal facet of the fibre using the ATM. This level of spatial mode control at the output of the fibre also suggests that more general point spread function engineering within the isoplanatic patch should be possible.

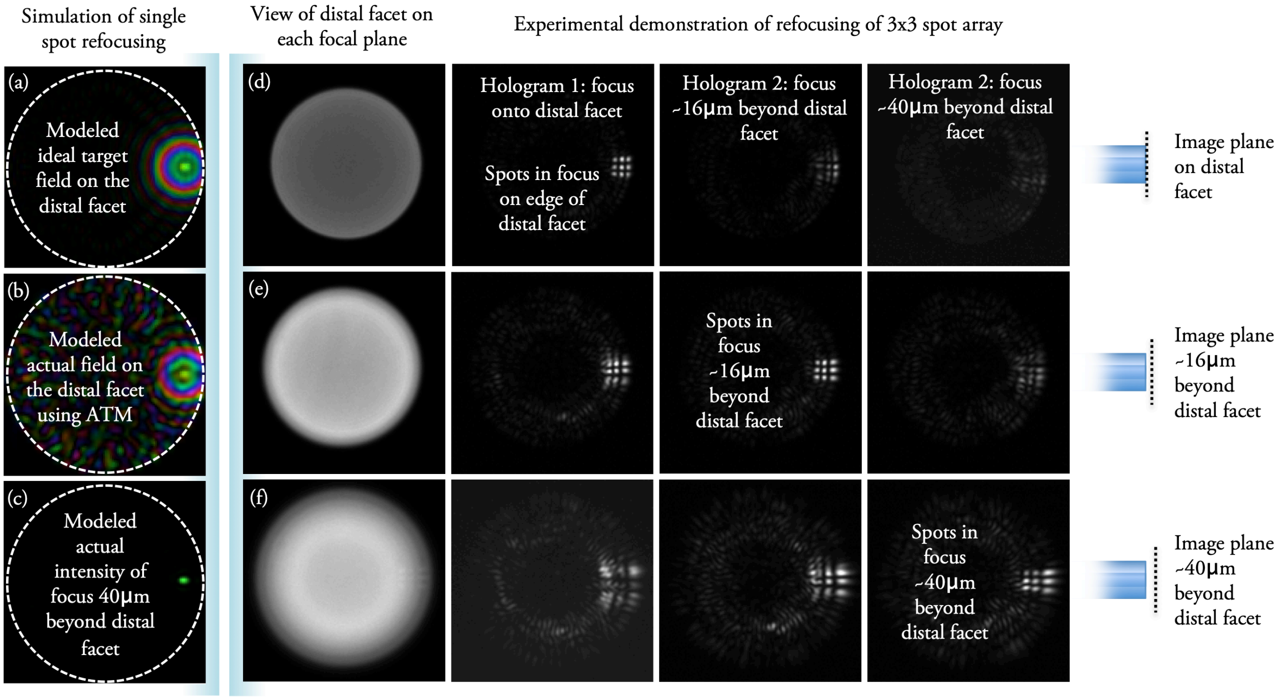

Supplementary Figure 5: **Focusing beyond the end of the distal facet**: (a-c) Simulation of the performance of axially refocusing a spot beyond the distal facet of the fibre. (a) The idea field required at the distal facet to create a focus 40 μm beyond the distal facet. (b) Actual field created at the distal facet when using the ATM. (c) Field in (b) has been numerically propagated 40 μm , and the intensity of the resulting focussed spot is shown. We see that the point is focussed more tightly in the vertical direction due to the shape of the isplanatic patch. (d-f) Experimental demonstration of refocusing a 3×3 array of spots. Row (d) shows images of the distal facet in focus (left panel), and images of the intensity at the distal facet when 3 different focusing holograms are applied to the DMD. We see that the spots are in focus in the centre-left panel. In row (e) the spots are in focus in the centre-right panel, ~ 16 μm beyond the distal facet. In row (f) the spots are in focus in the right hand panel, ~ 20 μm beyond the distal facet. Note: in (f) the right hand column of spots at the very edge of the fibre become distorted as they field forming them is heavily restricted by the aperture of the fibre core. The brightness of the off-focus images have been enhanced to more clearly visualise the intensity patterns.

Supplementary Note 6: Pattern projection through guide-star calibrated MMFs

Once the ATM of the MMF has been estimated, it can be used to not only image through the fibre, but also project patterns through the fibre within the isoplanatic patch. Supplementary Figure 6 shows examples of patterns consisting of arrays of focussed spots at the distal facet, and comparisons with patterns produced when the full TM of the fibre is measured. We see that the fidelity of pattern projection within the isoplanatic patch using the ATM is not significantly lower than that achievable with a fully sampled TM. Notably, although the contrast is lower using the ATM, in both cases the focussed spots are of a similar beam waist, indicating that the resolution of imaging within the isoplanatic patch is similar to that of a fully sampled TM.

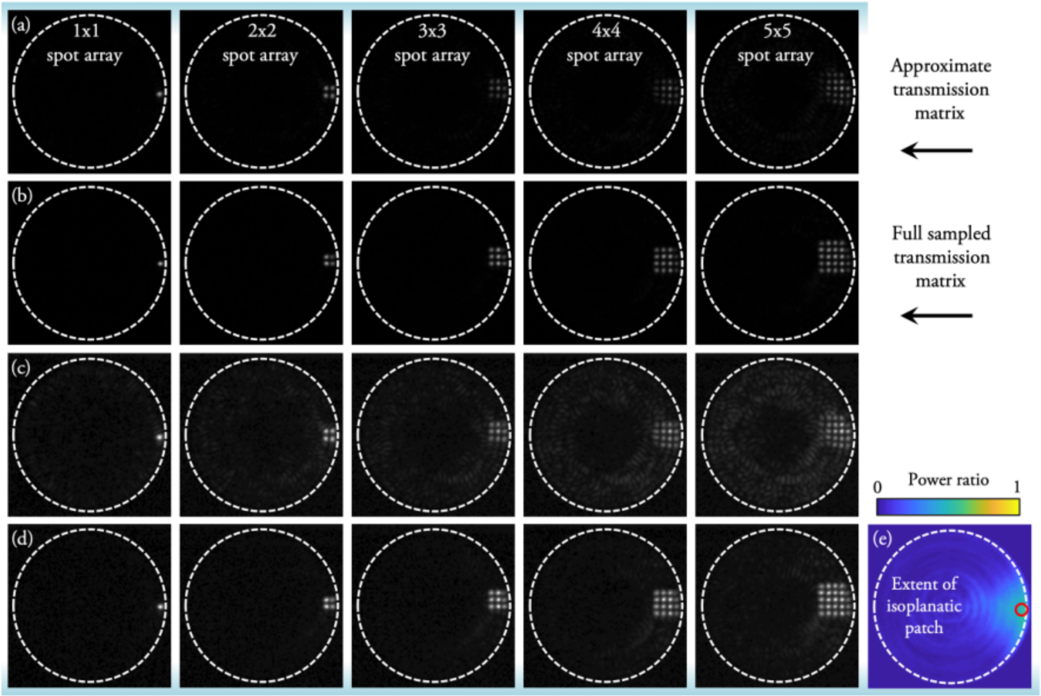

Supplementary Figure 6: **Pattern projection using the approximate transmission matrix (ATM)**: Row (a) shows experimental results projecting arrays of spots of increasing number through the MMF using the ATM. Row (b) shows the same arrays projected with the fully sampled TM for comparison. Rows (c) and (d) show the same results as (a) and (b) but with the contrast enhanced to highlight relative level of the background speckles in each case. (c) using ATM, (d) using fully sampled TM. (e) shows the power ratio of a single focussed spot (see main text for definition) as it is scanned over the distal facet - indicating the extent of the isoplanatic patch.

Supplementary Note 7: Focussing through deformed MMFs

Supplementary Figure 7 shows the performance of using a guide-star to focus through a deformed ~ 30 cm length of multimode optical fibre. Bending of a multimode optical fibre typically varies the optical path length of each PIM by a different amount, thus reducing the level of constructive interference at the target focus location on the distal facet. The guide-star then provides a way to measure these new optical path lengths to restore high fidelity focussing. Although able to mitigate phase changes, guide-star assisted imaging also requires that power on the diagonal (p_d) of the TM remains significant even when the MMF is bent. In our experiments we find that bending the fibre only results in a modest reduction in p_d and consequently only slightly reduces the contrast of focii that can be achieved. We note that in our proof-of-principle experiments we have estimated $\mathbf{P}$ based on a model of an ideal straight fibre, which results in a relatively low p_d of ~ 10-20% in all fibre configurations, and so an equivalently small isoplanatic patch in each case. In future applications we envisage first having the ability to rapidly measure the full TM of a MMF before it is deployed as a micro-endoscope. Therefore, we anticipate diagonalising this fully sampled measurement to obtain a high fidelity estimate of $\mathbf{P}$, which we believe would result in an isoplanatic patch that covered the majority of the output facet of the core, even if the fibre was bent.

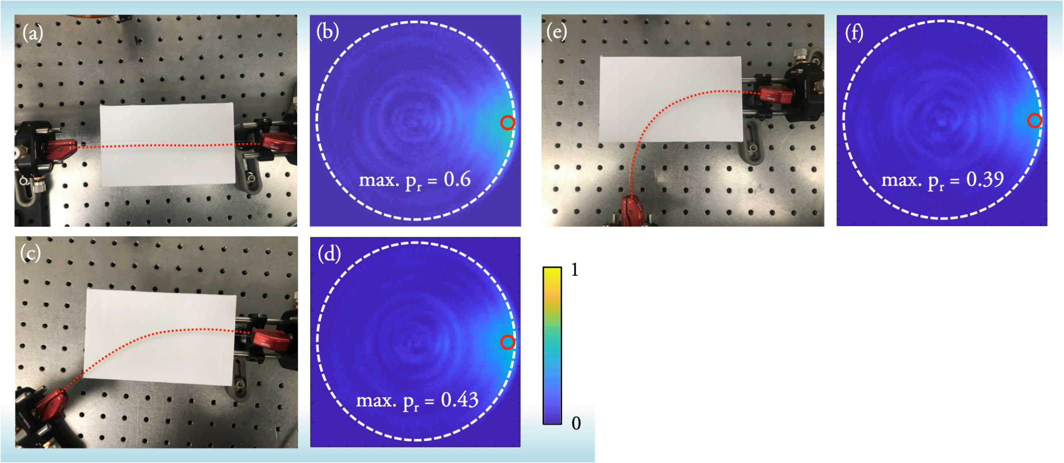

Supplementary Figure 7: **Focussing through deformed optical fibres**: (a-c) show pictures of the fibre configuration on the optical bench - optical fibre configuration is highlighted with a dashed red line. (a) straight fibre. (b) fibre bent through ~ 40 degrees, (c) fibre bent through ~ 90 degrees with a radius of curvature of ~ 10 cm. (d-f) show the corresponding power-ratio maps for each case.

Supplementary Note 8: Focussing onto a guide-star

In our proof of principle experiments, we emulate a guide-star by looking only at data from a single camera pixel. We now describe the focussing protocol were a real guide-star to be used. There are two approaches, depending upon the nature of the guide-star:

(i) Phase conjugation. If the guide-star is a highly reflective particle, then when light is transmitted through the fibre, we can assume that the return signal will be dominated by light reflected from the guide-star - as long as the distal field overlaps with the location of the guide-star. The spatial mode (amplitude and phase function) of this reflected light at the proximal facet of the fibre is the phase conjugate of the incident field required to focus onto the guide-star ($\mathbf{u}^{gs}$). It can be determined in a single shot (i.e. single camera image) using digital holography with a coherent reference beam. This method has the advantage that it is extremely fast and so can be performed in real-time. A disadvantage is that the return signal may also be mixed from light reflected from across the distal fibre facet, reducing the fidelity of the measurement of $\mathbf{u}^{gs}$.

(ii) Phase stepping holography. If the guide-star is a fluorescent particle, then in the returning signal we can separate any excitation light reflected from the fibre facet from fluorescence emitted by the guide-star by spectral filtering. However, we do not have access to a coherent reference beam with which to measure the phase of $\mathbf{u}^{gs}$ in a single shot. In this case we can recover $\mathbf{u}^{gs}$ in the following way: we sequentially inject a series of 'test' spatial modes that fully sample the modes supported by the fibre, along with a coherent reference beam that overlaps with the location of the guide-star. We measure the total excited fluorescent signal transmitted back to the proximal end for each input - which may be achieved using a fast photodiode. This return signal is directly proportional to the excitation intensity at the guide-star location. By varying the relative phase between the reference and the nth injected mode, the return fluorescent signal encodes both the amplitude and phase of input mode n at the guide-star location, in the same way as described in Sec. S7 above. Although this method is slower that (i), it may still be performed rapidly using high speed DMDs to modulate input patterns.

Supplementary Note 9: Estimation of loss in the optical system.

Here we estimate the level of loss in the optical system.

DMD loss: This is the major source of loss in our optical system. We choose to use a DMD to shape input light fields as DMDs offer a high modulation rate (up to ~20kHz) and have been shown to create fields of high fidelity (see ref. [4]). However, the binary amplitude modulation of DMDs means that these advantages come at the expense of low light conversion efficiency. Accounting for diffraction from the pixels themselves (which create multiple additional diffraction orders), and the inherently lossy nature of amplitude and phase modulation, we estimate that ~1% of the incident power is transmitted into the target beam.

Insertion loss: The numerical aperture of the light focused into the fibre is set to slightly overfill the NA of the fibre, thus resulting in some insertion loss during the measurement of the TM. The fibre in our experiment is not antireflection coated, and so incident light, travelling from air medium to glass suffers an additional ~4% reflection loss on the input to the fibre. These losses could be mitigated by matching the NA of the incident light to that of the fibre during TM measurement and using an antireflection coated fibre.

Mode dependent loss: PIMs of higher mode indices possess more power propagating close to the critical angle of total internal refection as they propagate through the MMF, and so are more likely to lose power into the cladding due to fibre

imperfections. However as our intended micro-endoscope application uses short ~30cm lengths of fibre, we expect this loss to be minimal. However, residual misalignments of the input will artificially increase the apparent mode dependent loss - as an inaccurately estimated PIM basis will result in inaccurately shaped modes launched into the fibre, and the modes of higher mode index lose extra light into the cladding. In the future this can be rectified by using optimization to precisely align the input to the fibre, as demonstrated in refs. [3, 5].

Supplementary References

[1] F. W. J. Olver, A. B. Olde Daalhuis, D. W. Lozier, B. I. Schneider, R. F. Boisvert, C. W. Clark, B. R. Miller, B. V. Saunders, H. S. Cohl, and M. A. McClain, eds. *NIST Digital Library of Mathematical Functions*, Release 1.0.26, (2020).

[2] Tomáš Čižmár, Michael Mazilu, & Kishan Dholakia. In-situ wavefront correction and its application to micromanipulation. *Nature Photonics*, 4(6):388, (2010).

[3] Martin Plöschner, Tomáš Tyc, and Tomáš Čižmár. Seeing through chaos in multimode fibres. *Nature Photonics*, 9(8):529, (2015).

[4] Sergey Turtaev, Ivo T Leite, Kevin J Mitchell, Miles J Padgett, David B Phillips, and Tomáš Čižmár. Comparison of nematic liquid-crystal and DMD based spatial light modulation in complex photonics. *Optics express*, 25(24):29874–29884, (2017).

[5] Maxime W Matthès, Yaron Bromberg, Julien de Rosny, & Sébastien M Popoff. Learning and avoiding disorder in multi-mode fibers. *arXiv preprint arXiv:2010.14813*, (2020).
