# Supplementary material for: Memory effect assisted imaging through multimode optical fibres
Source: Nat Commun. 2021 Jun 18;12:3751. doi: 10.1038/s41467-021-23729-1 (PMC8213736; doi:10.1038/s41467-021-23729-1)
Supplement: Supplementary file 3 — Description of Additional Supplementary Files [file 41467_2021_23729_MOESM3_ESM.pdf]

Description of Additional Supplementary Files

Supplementary Movie 1: Fully Sampling the TM of an MMF

Description: This movie shows the input and output fields that fully sample the TM of an MMF. It depicts experimentally measured complex fields excited at the distal facet of the MMF as an incident focused spot is scanned across the input facet. The left-hand panel shows the position of the incident focused spot on the proximal facet. The right-hand panel shows the resulting complex field excited at the distal facet. Here brightness is proportional to the amplitude of the field, and the colour is proportional to the phase of the field, using the same scale-bar as shown in Supplementary Fig. 4. This movie highlights several features of the MMF optical transform: (1) the rotational memory effect can be seen as the distal field appears to rotate when the scanning focus approximately tracks an azimuth on the input facet. This rotation is first anti-clockwise, and later clockwise, depending on when the scanned focus is on the left- or righthand side of the central fibre axis. (2) The general structure of the output field depends on the radial position of the input focus. The distal output field forms a bright speckled ring, the intensity of which is maximised at the same radius as the input spot. (3) The average speckle grain size increases as the radial position of the input spot decreases. Points (2) and (3) are due to the excitation of a different subset of the PIMs depending upon the radial position of the input spot. PIMs must overlap with the input spot position to be excited, which in turn produces the bright ring at the same radius at the output facet of the MMF. When the input spot moves closer to the central fibre axis, lower order PIMs are preferentially excited, which have lower transverse k-vectors, thus resulting in the output field exhibiting larger speckle grains.

Supplementary Movie 2: Scanning a focus over the distal facet isoplanatic patch

Description: This movie shows an experimental measurement of the intensity of the focus, created using the ATM, and scanned across the isoplanatic patch. The field-of-view covers the 50µm diameter MMF core, the boundary of which is marked with a white hatched circle. The position of the guide-star is marked with a red circle (centre-top). We see the spot can be scanned in 2D, and its power-ratio decreases as it is moved away from the guide-star location, as discussed in the main text.
